# Inhibitory Receptor Expression Depends More Dominantly on Differentiation and Activation than “Exhaustion” of Human CD8 T Cells

**DOI:** 10.3389/fimmu.2013.00455

**Published:** 2013-12-19

**Authors:** Amandine Legat, Daniel E. Speiser, Hanspeter Pircher, Dietmar Zehn, Silvia A. Fuertes Marraco

**Affiliations:** ^1^Clinical Tumor Biology and Immunotherapy Unit, Department of Oncology, Ludwig Center for Cancer Research, Lausanne University Hospital (CHUV), Lausanne, Switzerland; ^2^Department of Immunology, Institute of Medical Microbiology and Hygiene, University of Freiburg, Freiburg, Germany; ^3^Swiss Vaccine Research Institute (SVRI), Epalinges, Switzerland; ^4^Division of Immunology and Allergy, Department of Medicine, Lausanne University Hospital (CHUV), Lausanne, Switzerland

**Keywords:** inhibitory receptors, CD8 T cell, activation, differentiation, T cell exhaustion

## Abstract

Under conditions of chronic antigen stimulation, such as persistent viral infection and cancer, CD8 T cells may diminish effector function, which has been termed “exhaustion.” Expression of inhibitory Receptors (iRs) is often regarded as a hallmark of “exhaustion.” Here we studied the expression of eight different iRs by CD8 T cells of healthy humans, including CTLA-4, PD1, TIM3, LAG3, 2B4, BTLA, CD160, and KLRG1. We show that many iRs are expressed upon activation, and with progressive differentiation to effector cells, even in absence of long-term (“chronic”) antigenic stimulation. In particular, we evaluated the direct relationship between iR expression and functionality in CD8 T cells by using anti-CD3 and anti-CD28 stimulation to stimulate all cells and differentiation subsets. We observed a striking up-regulation of certain iRs following the cytokine production wave, in agreement with the notion that iRs function as a negative feedback mechanism. Intriguingly, we found no major impairment of cytokine production in cells positive for a broad array of iRs, as previously shown for PD1 in healthy donors. Rather, the expression of the various iRs strongly correlated with T cell differentiation or activation states, or both. Furthermore, we analyzed CD8 T cells from lymph nodes (LNs) of melanoma patients. Interestingly, we found altered iR expression and lower cytokine production by T cells from metastatic LNs, but also from non-metastatic LNs, likely due to mechanisms which are not related to exhaustion. Together, our data shows that expression of iRs *per se* does not mark dysfunctional cells, but is rather tightly linked to activation and differentiation. This study highlights the importance of considering the status of activation and differentiation for the study and the clinical monitoring of CD8 T cells.

## Introduction

Inhibitory co-receptors (iRs) encompass a group of molecules that function within the immune synapse to inhibit T cell function, opposite to co-stimulatory receptors (1). Over the last decade, several iR members have been closely linked with the phenomenon of T cell “exhaustion,” including PD1, CTLA-4, TIM3, LAG3, CD160, and 2B4 (2–6). “Exhaustion” of T cells terms the gradual reduction of cellular functions that occurs in CD8 T cells in conditions of chronic antigen exposure. This has been first reported in the prototypic LCMV mouse model of chronic infection with LCMV clone 13, with many findings confirmed in other chronic infections and cancer, in mice and in patients (HIV, HCV, EBV in SLE) (2–11).

Current models link the “exhaustion” phenotype to a particular over-expression of several iRs, which are considered key to the molecular signature of exhausted cells and are proposed to co-regulate CD8 T cell exhaustion (4, 6, 8, 12, 13). As a consequence, iRs are often referred to as “exhaustion markers” (14–16). In previous studies, we observed that CD8 T cells in melanoma metastases showed reduced function (low cytokine production) and high level expression of several iRs, reminiscent of exhausted cells (5, 17). However, further analysis of iR expression in human CD8 T cells from melanoma patients and healthy individuals also showed that iR profiles can change depending on the differentiation status, anatomical localization, and antigen-specificity (18).

Positive expression of iRs such as PD1, CTLA-4, TIM3, LAG-3, 2B4, CD160, and BTLA has been directly linked to reduced cytokine production by T cells from cancer patients (including reports from our group) or in the chronic LCMV mouse model of “T cell exhaustion” and other viral infections (7, 13, 19–23). However, recent studies reported that PD1 expression in healthy donors does not correlate with lower functionality, but rather correlates with differentiation to effector memory phenotype (18, 24). And recently, we showed that PD1 positive CD8 T cells in peripheral blood mononuclear cells (PBMC) of healthy donors and melanoma-specific CD8 T cells in PBMC of melanoma patients are not necessarily functionally impaired (25).

While there is increasing evidence that the expression of iRs can vary depending on activation and differentiation, this has not been yet thoroughly investigated for several different iRs in human CD8 T cells (1, 26). Critically, in view of the current assumption that expression of iRs indicates “T cell exhaustion,” it must be determined whether this equation holds true in general, including both pathological and healthy contexts, or whether it is primarily applicable to conditions of chronic antigen exposure.

In the present study, we aimed to determine the significance of the expression of a broad panel of iRs in human CD8 T cells, and investigate whether expression of iRs was primarily associated with T cell dysfunction. To this end, we performed a thorough analysis of iR expression in CD8 T cells from healthy individuals, during the course of stimulation with anti-CD3 and anti-CD28 antibodies (aCD3/aCD28), and taking into consideration the differentiation status of the cells. Based on our previous studies and on the reported molecular signature of T cell exhaustion (2–5, 7, 8, 12, 27), we focused on the following eight iRs: PD1 (and its ligand PDL1), CTLA4, LAG3, TIM3, CD160, 2B4, KLRG1, and BTLA. We investigated the dynamics of expression of several iRs as well as possible correlations with increased or decreased cytokine production. In addition, blood-derived CD8 T cells were compared to CD8 T cells from metastatic and normal lymph nodes (LNs) of melanoma patients. Conclusively, we find that iR expression is predominantly impacted by the differentiation as well as the activation status of the cells, with more modest changes observed in both normal and metastatic LNs. More strikingly, CD8 T cells that are positive for iRs are not necessarily less capable of cytokine production, but rather, expression of certain iRs can positively correlate with differentiation status and with several standard activation markers.

## Materials and Methods

### Peripheral blood and lymph node samples

Peripheral blood from healthy donors was obtained from leukocyte-rich preparations provided by the Blood Transfusion Center of Lausanne, Switzerland. Peripheral blood as well as metastatic [tumor-infiltrated lymph node (TILN)] and non-metastatic (normal) LNs were obtained from melanoma patients following surgery and upon written informed consent based on the study protocol approved by the ethical commission of the University of Lausanne. The material originated from the following patients: LAU309 (PBMC, TILN), LAU478 (PBMC, two non-metastatic LNs), LAU1127 (PBMC, three non-metastatic LNs), LAU1299 (PBMC, TILN), and LAU1413 (PBMC, 2 TILNs). Cell suspensions of LN samples were prepared mechanically, in absence of digestion enzymes, after finely mincing surgery specimens with scissors directly in complete medium (composition described below). PBMC from healthy donors and patients were obtained following density gradient fractionation of blood samples using Lymphoprep™. All samples were immediately cryopreserved in RPMI 1640 supplemented with 40% FCS and 10% DMSO until the experiment was performed.

### Cell culture and stimulations

The Complete Medium used was RPMI 1640, complemented with 10% heat-inactivated FCS, 1% non-essential aminoacids (Gibco), 1% l-glutamine (Gibco), Hepes (10 mM), and 10,000 U/ml of penicillin/streptomycin (Gibco). Of note, no cytokines nor growth factors were added. For short-term stimulations (up to 24 h), CD8 T cells were stimulated in isolation. Where indicated, isolated CD8 T cells were obtained by positive selection using the Dynabeads^®^ Human CD8+ selection (Invitrogen), according to the manufacturer’s protocol except the use of anti-human CD8 antibody at 30% to minimize co-purification of CD8+(^int^) NK cells. For longer-term treatments (>24 h), total PBMC were used given the susceptibility of isolated CD8 T cells in culture in absence of stimuli (=medium controls). No exogenous IL-2 was used in the cultures and stimulations in this study. N.B. unlike CD4 T cells, isolated CD8 T cells do not produce IL-2 in absence of stimulus and therefore addition of exogenous IL-2 is required for the survival of isolated CD8 T cells longer than 24 h (particularly “medium” controls); in contrast, whole PBMC cultures do not require exogenous IL-2. Isolated CD8 T cells or PBMC were cultured at a density of 0.75–1 × 10^6^ cells/ml/cm^2^ in flat-bottom plates. For experiments using patient-derived CD8 T cells (of limited availability), U-bottom plates were used for stimulations were less than 50,000 purified CD8 T cells were available. In experiments using LN-derived CD8 T cells, total cell suspensions (from TILN, normal LN, or PBMC) were allowed to rest overnight in culture in complete medium (without IL-2), prior to CD8 T cell isolation and the 4 h stimulation assay. Anti-CD3 and anti-CD28 (aCD3/aCD28) beads were prepared using the “T cell expansion” kit (Miltenyi Biotec), coated with the antibodies according to the manufacturer’s protocol. Stimulation with anti-CD3 and anti-CD28 beads was performed at a 1:1 ratio with cells. When several time-points were assessed, reverse kinetics were used, treating cells at different times before the simultaneous analysis of all samples. For intracellular staining of cytokines as well as CTLA-4, Brefeldin A was added at 10 μg/ml during the last 4 h of culture. For CD107a staining, the antibody was added to the cultures during the last 4 h of stimulation.

### Flow cytometry

Data acquisition was performed with a Gallios flow cytometer (Beckman Coulter, 3-laser configuration) with antibody panels limited to 10-colors. The data was processed with FlowJo (Tree Star Inc., v9.5.2) software and co-expression analyses were obtained using SPICE software (v5.3) (28). The anti-human antibodies used were anti-CD8 (APC-Alexa750, clone B9.11, Beckman Coulter), anti-CCR7 (Brilliant Violet, clone G043H7, Biolegend), anti-CD45RA (ECD, clone 2H4LD11LDB9, Beckman Coulter), anti-CD16 (Krome Orange, clone 3G8, Beckman Coulter), anti-TNFa (intracellular, Alexa700, clone Mab11, BD Biosciences), anti-IFNg (intracellular, PE-Cy7 and PE with clone 4SB3, APC with clone B27, BD Biosciences), anti-IL-2 (intracellular, PerCP-Cy5.5, clone MQ1-17H12, BD Biosciences), anti-Granzyme B (intracellular, FITC, clone GB11, Biolegend), anti-CD107a (PE, BD Biosciences, clone H4A3), anti-human PD1 (PerCP-eF710, clone eBioJ105, eBioscience), anti-human PDL1 (PE-Cy7, clone MIH1, eBioscience), anti-CTLA-4 (intracellular, PE or APC, clone BNI3, BD Biosciences), anti-TIM3 (PE, clone 344823, RnD), anti-LAG3 (Alexa488, clone 17B4, AbD Serotec), anti-CD160 (Alexa647, clone BY55, eBioscience), anti-2B4 (PE-Cy5.5, clone C1.7, Biolegend), anti-KLGR1 (A488, clone 13F12F2, provided by H. Pircher), anti-BTLA (PE-Cy7, clone BTLA7.2, Beckman Coulter, or PE, clone J168-540, BD Biosciences), anti-4-1BB (intracellular, PE-Cy7, clone 4B4-1, Biolegend), anti-CD25 (PE-Cy7, clone BC96, Biolegend), anti-CD38 (Alexa700, clone HIT2, eBioscience), and anti-CD69 (FITC, clone FN50, BD Biosciences).

All steps were performed at 4°C. Surface staining was performed using antibodies in FACS buffer (PBS with 5 mM EDTA and 0.2% BSA) for 30′. Dead-cell exclusion was done after surface staining using the fixable dead-cell marker Vivid-Aqua (Molecular Probes^®^, Invitrogen). Cells were fixed overnight in 1% formaldehyde. Intracellular staining was performed using antibodies in FACS buffer with 0.1% saponin for 30′ at 4°C.

Of note, conditions for the detection of the various makers (iRs and activation) were optimized relative to intracellular versus surface staining, as well as presence or absence of Brefeldin A (necessary for cytokine intracellular retention and staining) (data not shown). CTLA-4 detection is optimal when performed intracellularly and is enhanced by Brefeldin A treatment of the cells in culture. Brefeldin A abrogates *de novo* CD69 surface expression but does not affect previously existing surface CD69 (i.e., it is possible to add Brefeldin A during the last 4 h of culture in mid- to long-term stimulations). 4-1BB is efficiently detected on the surface of activated cells; however, presence of Brefeldin A abrogates surface 4-1BB and imposes its intracellular staining.

### Quantifications and statistical analyses

Quantifications were made based on the softwares FlowJo, Graphpad Prism, and SPICE. For each marker, the analysis was based on visible positive and negative populations, and isotype-matched controls were used to verify positivity and used to set the gates (isotype samples were set <1% positive, with 1% considered background staining). For the analysis of cytokine production within iR positive cells versus iR negative counterparts, only populations >3% where considered; e.g., LAG-3 positive cells were not analyzed; nor Naïve cells that are PD1 positive or EMRA cells that are 2B4 negative (populations equal or below 3% are marked as NA, not applicable). For statistical comparison of pie charts, the built-in test in SPICE software (v5.3) was used (using 10,000 permutations) (28); other *p*-values were obtained using the statistical tests as detailed in the figure legends, with ns, not significant; *p < 0.05; **p < 0.01; ***p < 0.001. Where shown, error bars indicate standard error of the mean.

## Results

### Inhibitory receptor (iR) expression in human CD8 T cells is inherently different in Naïve versus differentiated subsets

**Figure 1:**
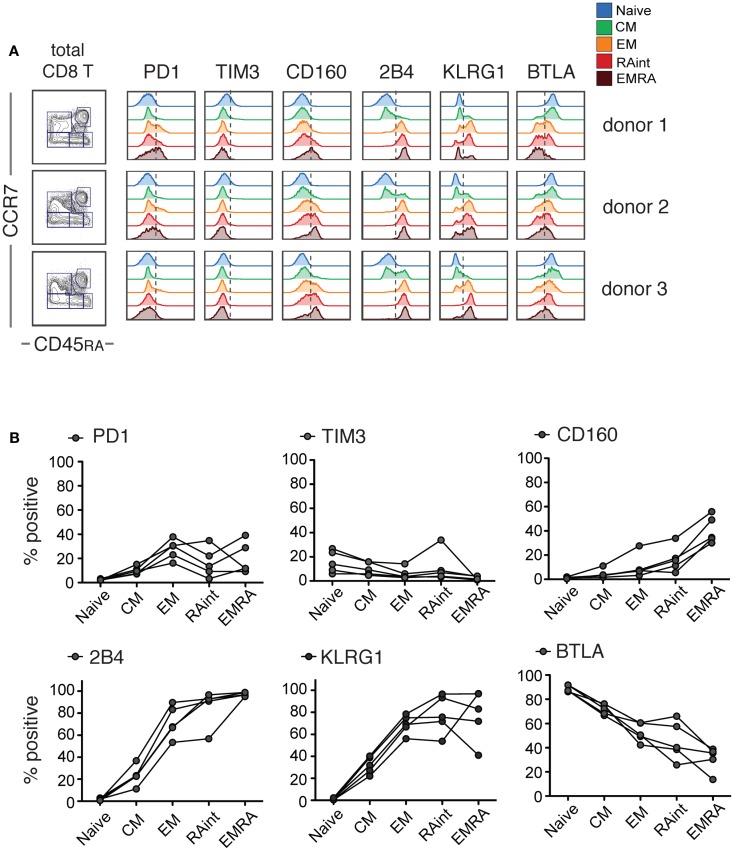
**IR profiles change with CD8 T cell differentiation**. Resting CD8 T cells isolated from healthy donor PBMC were analyzed for the indicated inhibitory receptors (iRs) (these samples correspond to the medium controls from the experiments in Figure 2). Three examples are shown. **(A)**. CCR7 versus CD45RA-based differentiation plot of total CD8 T cells and off-set overlay histograms for each iR comparing the differentiation subsets, gated as shown in detail in Figure S1 in Supplementary Material. Three representative donors are shown. **(B)**. Quantification of A shown as percentage positive within each subset (*N* = 5).

For a detailed investigation of iR expression and correlations with cytokine production, we used CD8 T cells isolated from PBMC of healthy individuals. Based on CCR7 and CD45RA markers, we identified Naive (CCR7+ CD45RA+), CM (CCR7+ CD45RA−), and effector cells (CCR7−). The latter were split into Effector Memory (EM CD45RA−), RAint (CD45RAint), or EMRA (EM CD45RA+) cells (gating strategy detailed in Figure S1 in Supplementary Material). We found that several iRs were particularly upregulated with differentiation: PD1, 2B4, KLRG1, CD160, and to a lesser extent, TIM3 (Figure 1). The opposite was seen for BTLA, which was predominantly expressed by Naive cells. Notably, there can be considerable differences amongst different donors (Figure 1B). Nevertheless, our data clearly show strong differences of iR expression depending on the differentiation status of human CD8 T cells, in agreement with previous studies (18, 23, 24).

### T cell activation can induce drastic changes in iR expression in human CD8 T cells

We then performed a kinetic study, in which we activated purified CD8 T cells with aCD3/aCD28 beads for various time-points, and analyzed iR expression in combination with IFNg and TNFa as functional readout.

**Figure 2:**
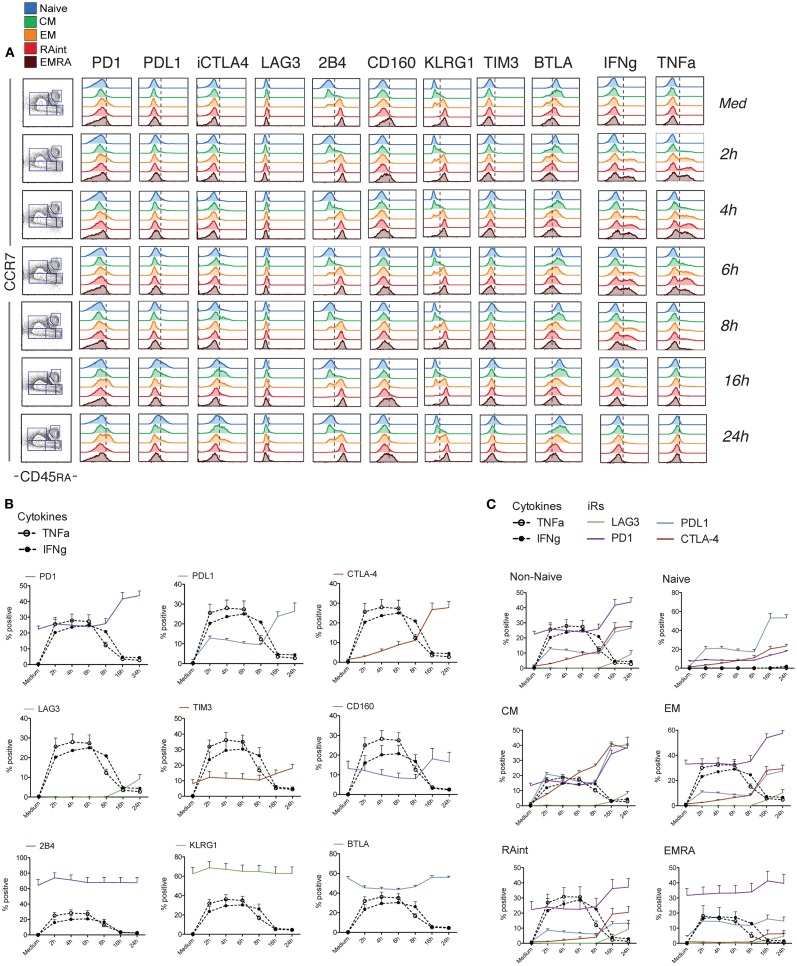
**Dynamics of iR expression relative to the cytokine production wave within 24 h of stimulation**. CD8 T cells isolated from healthy donor PBMC were analyzed at different time-points after stimulation with anti-CD3 and anti-CD28 antibodies and analyzed for the various iRs together with TNFa and IFNg, as indicated (*N* = 5). **(A)**. One representative example of the analysis, done as in Figure 1A. **(B)**. Quantification showing the percentage positive cells for each iR, together with IFNg and TNFa, within total non-naive CD8 T cells (NB. Naive cells do not produce cytokines – Figure S2 in Supplementary Material). **(C)**. Grouped quantification for the iRs that showed up-regulation, in Non-Naive cells or the various differentiation subsets as indicated.

Of note, only non-naive cells showed co-expression of multiple effector molecules, including Granzyme B, IFNg, TNFa, IL-2, and CD107a translocation upon stimulation (Figure S2 in Supplementary Material). Cytokine production occurred within the first few hours of stimulation, with a peak at 2–6 h. Concomitant analysis of the expression of iRs in Non-Naive cells showed that activation induced clear changes already early on: CTLA-4, LAG3, and the ligand PDL1 were absent in resting cells (medium control) but were clearly upregulated with activation, while PD1 and BTLA were already present and modulated up (PD1) or down (BTLA) during activation (Figure 2). In contrast, the levels of the other receptors (KLRG1, 2B4, CD160, and TIM3) remained stable within the 24 h of stimulus. When the various subsets were analyzed separately (Figure 2C; Figure S3 in Supplementary Material), the extent of up-regulation of iRs varied depending on the differentiation, with CM cells showing more marked changes and EMRA cells showing rather stable expression. Hence, changes were stronger in early differentiation stages, where iRs were low in the resting state.

**Figure 3:**
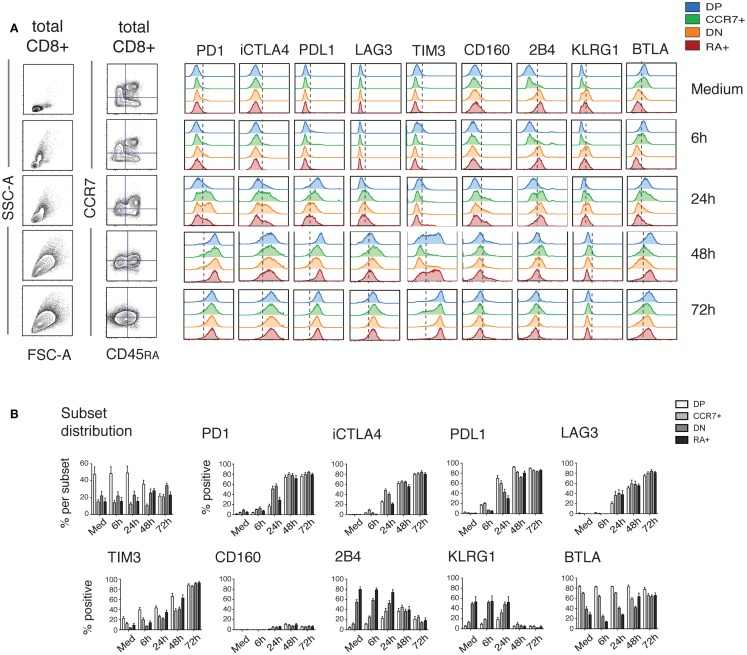
**Dynamics of iR expression over 3 days of stimulation**. PBMC from healthy donors were stimulated for different time-periods with anti-CD3 and anti-CD28 antibodies. CD8 T cells were analyzed similarly to Figure 1 for the various iRs. **(A)**. CCR7 versus CD45RA-based differentiation plot and FSC versus SSC plot of total CD8 T cells, as well as off-set overlay histograms for each iR comparing the various subsets as indicated. In order to analyze iRs during the development of an activated blasting population, we used a constant quadrant gating, based on the medium sample, for CCR7 and CD45RA. Despite the fact that the gating is obsolete by 72 h, this highlights the development of a homogeneous blast expressing various iRs. One representative example is shown. **(B)**. Quantification showing the percentage positive cells for each iR (*N* = 5).

Intriguingly, the changes in iR expression were more marked at the later time-points, when the cells stopped cytokine production (from 16 h) (Figure 2). Therefore, we next extended the kinetics of activation up to 72 h (Figure 3). Indeed, several iRs were strongly upregulated. In correlation with the development of a homogeneous blast population of activated CD8 T cells (CD45RA− CCR7−), by 72 h, cells uniformly expressed high levels of PD1 and its ligand PDL1, CTLA-4, LAG3, and TIM3, while they lost expression of 2B4 and KLRG1, and remained low for CD160. Only BTLA showed variable levels. Thus, CD8 T cell activation can drastically change the levels of certain iRs, albeit with varying kinetics and magnitude.

### Expression of iRs does not always indicate lower T cell function but rather correlates with T cell differentiation

**Figure 4:**
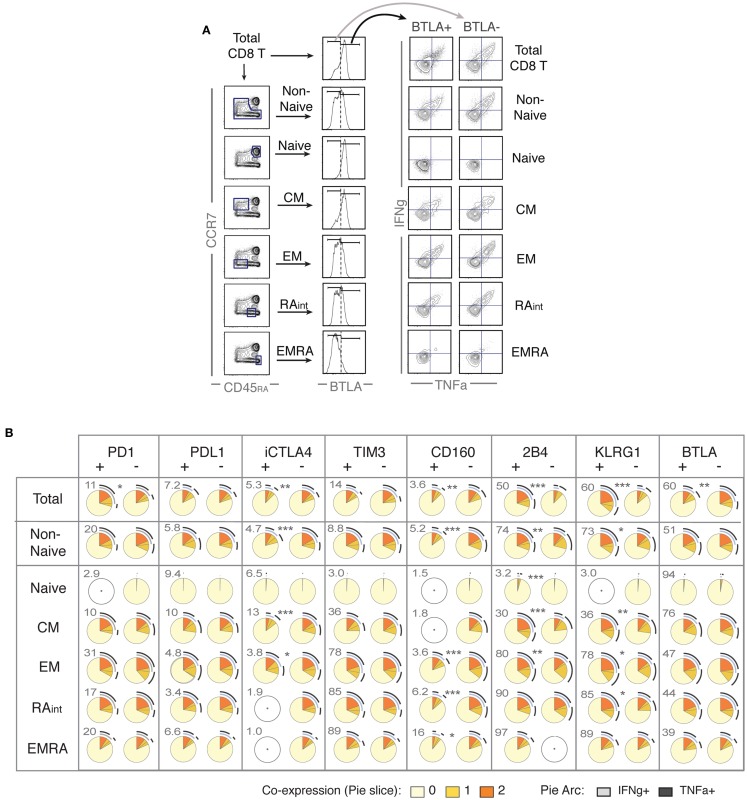
**Positive expression of iRs does not necessarily mark lower cytokine production but can rather correlate with T cell differentiation**. CD8 T cells from healthy donors were stimulated with anti-CD3 and anti-CD28 beads for 4 h, similarly as in Figure 2 (*N* = 15). **(A)**. Analysis strategy to compare iR positive versus negative cells, depending on CD8 T cell differentiation. Total CD8 T cells or each differentiation subset (as indicated) was split into iR positive and iR negative cells, which were subsequently analyzed for cytokine production. **(B)** Pie charts showing the cytokine production in iR positive or negative cells, considering either in total CD8, in Non-Naïve, or in each of the differentiation subsets, as indicated in each row. Pie arcs and slices represent the percentage positive for IFNg and/or TNFa, as indicated in the legend. The numbers adjacent to the iR positive pies, above and to the left of each pie, indicate the % of iR positive cells within the differentiation gate considered. An empty gray circle means the population does not apply (i.e., only populations representing iR expression gates >3% were considered). The *p*-values shown apply to the comparison of iR positive versus iR negative counterparts (indicated in between pairs of pies that showed significance).

In the light of the general concept that iR expression indicates “exhaustion,” we investigated the potential of iR positive cells to produce cytokines. Further to the changes in iR expression observed during stimulation of CD8 T cells (Figures 2 and 3), the focus of our study was therefore to determine whether iR positive CD8 T cells are less functional than their iR negative counterparts; whether positive iR expression marks dysfunctional CD8 T cells. To address this, we split populations into iR positive cells and iR negative counterparts, and subsequently quantified IFNg and TNFa production within each iR positive or negative group. In addition, we distinguished various populations, including total CD8 T cells, non-naive cells, or each individual differentiation population (Figure 4A).

The analysis on IFNg and/or TNFa production is shown using pie charts (SPICE-based, Figure 4B) or total cytokine-producing cells (Figure S4 in Supplementary Material). Crucially, we found very distinct results depending on the population considered. For instance, considering total CD8 T cells, BTLA positive cells clearly produced less cytokines than BTLA negative cells. However, this difference was greatly diminished when only non-naive cells were taken in consideration, and there was no difference between BTLA positive or BTLA negative cells when each individual differentiation subset was analyzed separately. The opposite observation was true for KLRG1 and 2B4, which strongly and positively correlated with higher cytokine production in total cells, but again these differences were largely diminished or absent within individual differentiation subsets. Conversely, iR negative cells appeared less cytokine-productive only when particular subsets were considered such as CM for CTLA-4. Although the expression of CD160 is generally very low (except in EMRA), it clearly marked less functional cells particularly in the EM/RAint subset. For PD1 positive versus negative cells, there were no major differences observed, except in the consideration of total cells, where PD1 positive cells were slightly more functional (only significance observed), which again rather correlates with the fact that PD1 is mostly expressed by more differentiated subsets (that produce more cytokines). Within the various differentiation subsets, only a trend was seen toward less cytokine in PD1 positive cells (Figure S4 in Supplementary Material). These results confirm previous observations for PD1 positive cells in healthy donor CD8 T cells (24, 25), where no significant association between PD1 expression and cytokine production was found, and provide parallel new evidence for the remaining seven iRs that were hereby analyzed. Overall, cytokine production in iR positive versus negative cells largely changed depending on the consideration of differentiation subsets; and iR positive cells were not less functional than iR negative counterparts, except for CTLA4 in CM and for CD160. In addition, it is of interest to note that there was substantial inter-donor variability, as it appears particularly evident from the pairings between iR positive and negative counterparts shown in Figure S4 in Supplementary Material (considering total cytokine-producing cells).

### Several iRs strongly correlate with activation markers

**Figure 5:**
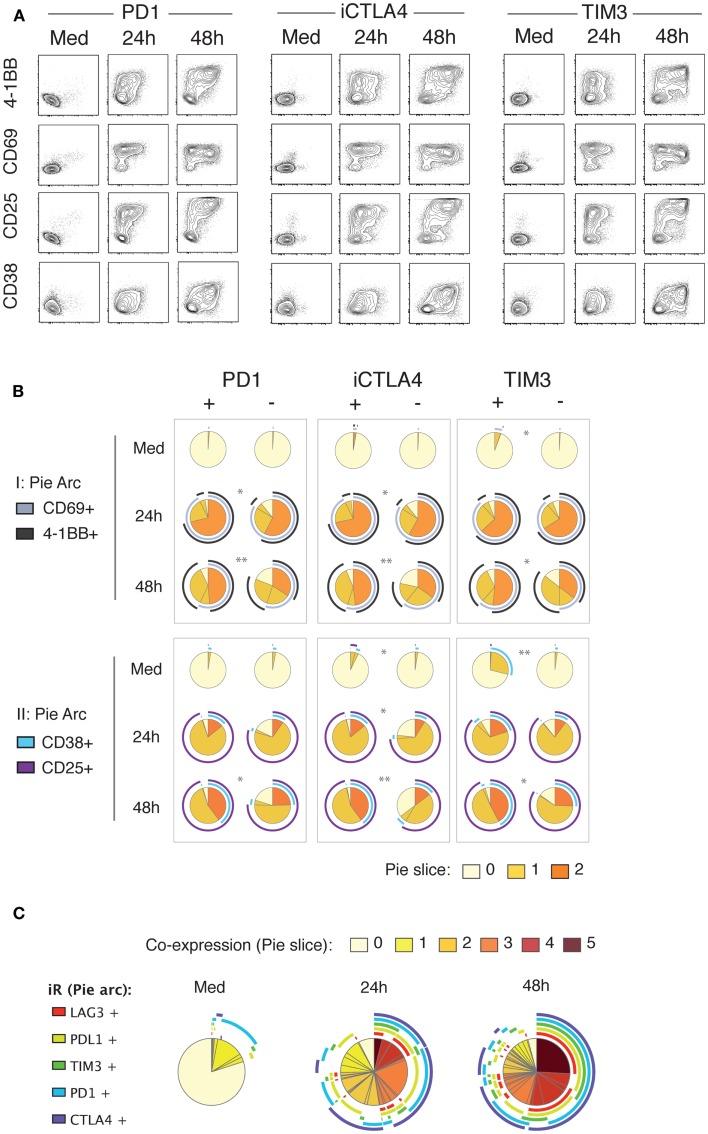
**Up-regulation of iRs during stimulation strongly correlates with activation markers**. Healthy donor PBMC were stimulated for 24 and 48 h with anti-CD3 and anti-CD28 antibodies. CD8 T cells were analyzed for co-expression of iRs and activation markers in two separate panels (I and II). **(A)**. Representative plot of PD1, CTLA4 or TIM3 (*x*-axis) versus the four activation markers as indicated, gating was on non-naive CD8 T cells. **(B)**. Quantification of the expression of activation markers in iR+ versus iR− counterparts (*N* = 5). **(C)**. Co-expression of PD1, CTLA4, TIM3, PDL1, and TIM3 on non-naïve CD8 T cells.

In view of the strong up-regulation of a number of iRs including PD1, CTLA4, TIM3, LAG3, and PDL1 with activation, and especially following the cytokine production wave, we sought to investigate the correlation between iR expression and activation markers. Strikingly, at 24 h of stimulation, up-regulation of PD1, CTLA4, and TIM3 clearly correlated with up-regulation of the activation markers 4-1BB, CD69, CD25, and CD38 (Figure 5). By 48 h, the co-expression of iRs and activation markers was more pronounced. Furthermore, iR+ CD8 T cells showed even more pronounced activation than their iR− counterparts (Figure 5B). Of note, PD1+, CTLA4+, and/or TIM3+ cells largely represent PDL1+ and LAG3+, all of which are co-expressed largely upon activation (Figure 5C). These data show that multiple iRs are co-expressed and positively correlated to T cell activation, suggesting that iR positivity could in fact indicate the cells that best responded to stimulation.

### Lymph node location, in addition to the tumor microenvironment, can influence the expression of iRs and cytokine production by CD8 T cells

The phenomenon of “T cell exhaustion” has been described in the particular context of chronic antigen stimulation. In cancer, it is primarily within the tumor microenvironment that CD8 T cells have been shown to over-express iRs, in correlation with lower cytokine production. Therefore, we assessed the relationship between iR positivity and cytokine production, taking in consideration the differentiation subsets, in human CD8 T cells isolated from metastatic LNs of melanoma patients (TILN). In parallel, as controls, we also isolated CD8 T cells from LNs of melanoma patients that were not infiltrated with melanoma cells (hereafter termed “non-metastatic LN” or “normal LN”). Isolated CD8 T cells from TILN and normal LN were directly compared with blood of healthy donors as well as blood from melanoma patients, in a 4 h assay with aCD3 and aCD28 beads.

**Figure 6:**
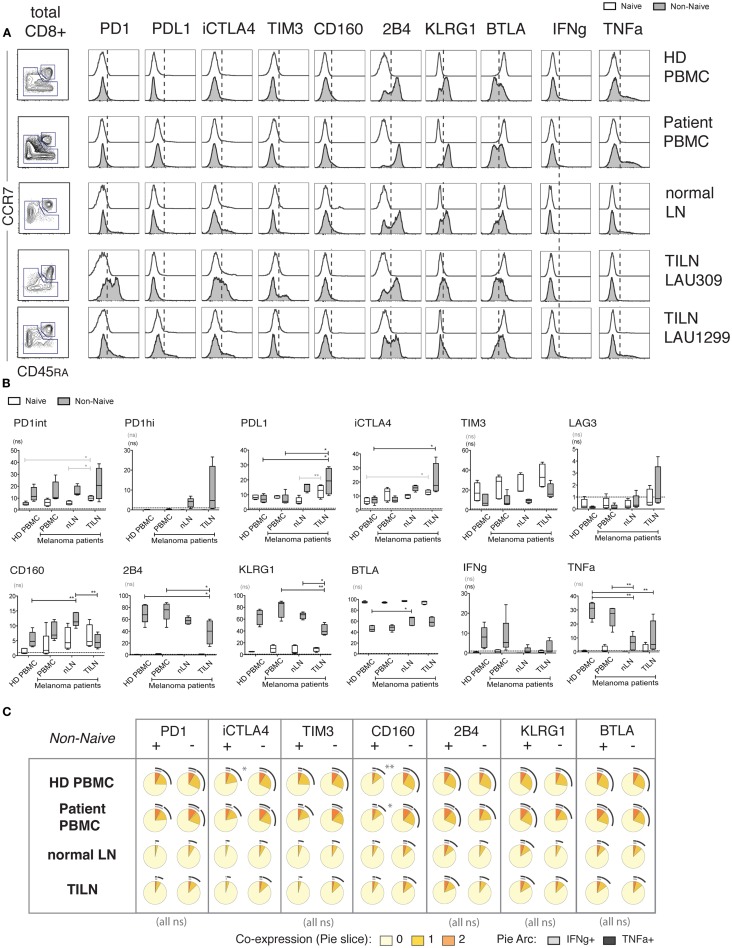
**Expression of iRs and cytokine production in human CD8 T cells from metastatic and non-metastatic lymph nodes differ from blood-derived T cells**. CD8 T cells isolated from PBMC of healthy donors (*N* = 5), PBMC of melanoma patients (*N* = 5), non-metastatic LN (“normal LN”) of melanoma patients (*N* = 5) and TILN (*N* = 4) were stimulated for 4 h with aCD3 and aCD28 beads. **(A)**. CCR7 versus CD45RA-based differentiation plot of total CD8 T cells, as well as off-set overlay histograms for each iR comparing Naïve and Non-naïve cells, as indicated. One representative example is shown for each, i.e., PBMC of healthy donor, PBMC of melanoma patient, and non-metastatic LN, and two distinct examples are shown for TILN. Naïve cells are shown in white histograms and non-naïve cells in gray histograms. **(B)**. Quantification showing the percentage positive cells for each iR as well as for IFNg and TNFa amongst the indicated sample groups. *p*-Values were based on multiple comparisons using one-way ANOVA with Bonferroni correction. **(C)**. Analysis of the expression of TNFa and IFNg in iR positive or negative cells, within non-naïve cells, amongst the indicated sample groups. Pie arcs and slices represent the percentage positive for IFNg and/or TNFa as indicated in the legend.

In contrast to the various subsets found in CD8 T cells from PBMC, LN samples displayed a dual subset distribution, including a naïve population and a non-naïve population; the non-naïve population was predominantly in the CM subset in normal LNs, and in CM or EM in TILN (Figure 6A; Figure S5A in Supplementary Material). For subsequent analysis, gating on non-naïve cells was performed because: (1) the various subsets found in blood-derived CD8 T cells could be missing in LNs, and (2) based on Figure 4, it is critical to distinguish at least naïve from non-naïve cells to compare the functionality of iR positive versus negative counterparts.

Within non-naïve cells, particularly high levels of iRs such as PD1, CTLA-4, and LAG-3, and decreased levels of KLRG1 and 2B4 were only found in some (but not all) TILN, in contrast to blood samples, and in agreement with previous reports (Figures 6A,B) (5). Of note, there was great variability in iR expression in TILN, where the sample showing lowest percentage of CM in non-naïve cells had higher PD1hi and CTLA-4 (Figures 6A,B; Figure S5B in Supplementary Material). Interestingly, our data shows that CD8 T cells from normal LNs may also display different iR expression as compared to blood-derived CD8 T cells, with notably the presence of PD1hi cells and increased levels of CTLA-4 (Figure 6B). Of note, this difference is not explained by the predominantly CM phenotype in LN samples (Figure S5B in Supplementary Material). Moreover, cytokine production was also low in non-naïve CD8 T cells from normal LNs, similar to TILN, and in contrast to blood-derived CD8 T cells (Figure 6B; Figure S5C in Supplementary Material). The fact that samples from normal LNs and TILN showed predominantly a CM phenotype could in part explain lower cytokine production as compared to non-naïve cells in blood (Figure S5C in Supplementary Material). However, cytokine production in LN samples still remained lower as compared to blood-derived CM cells (analysis not shown) and the% of CM did not correlate with cytokine production within the normal LN or TILN groups (Figure S5C in Supplementary Material), pointing toward other factors influencing lower cytokine production in LN samples.

Pertinently, in order to address the functionality of CD8 T cells that positively express iRs in the various anatomical locations, we focused on the question of comparing iR positive cells versus negative counterparts, and considering non-naïve cells (minimal requirement based on Figure 4). Importantly, associations between iR positive versus negative expression and cytokine production were relatively similar across blood or LN groups (Figure 6C); Nonetheless, in agreement with previous observations, PD1hi cells that were found uniquely in LN samples (and were absent in blood) showed reduced cytokine production as compared to PD1int or negative counterparts (Figure S6 in Supplementary Material) (19, 29). The overall results are described in Table 1 and further discussed below in more detail.

**Table 1:** **Summary on the expression of iRs and its link to cytokine production in CD8 T cells, considering differentiation, activation, and anatomical location**.

iR:	Changes of iR expression				iR+ cells show less cytokine production than iR− counterparts, considering:		
	With differentiation	With activation^a^	In normal LN^b^	In TILN^b^	Total cells (≈artifact^c^)	Subsets (i.e., corrected for differentiation)	Anatomical location
PD1	Increased (+ in effectors)	+++	Presence of PD1hi	Presence of PD1hi	Opposite!: IR+ = slightly more cytokines	No	Trend in LN
CTLA-4	(Absent in steady state)	++++	Increased	Increased	Yes (slightly)	Yes in non-naive/CM	Yes in HD PBMC; trend in all other locations
TIM-3	Increased in N and EMRA	+++	Similar	Can be increased	Trend	No	Trend in patient blood and TILN
LAG-3	(Absent in steady state)	++++	Similar	Can be increased	NA	NA	NA
CD160	Increased (+ in EMRA)	Stable	Increased	Similar	Yes (note very low fraction of iR+)	Yes	Yes in blood; trend in LN
2B4	Increased (progressive+)	Stable or –	Similar	Decreased	Opposite!: IR+ = more cytokines	No (iR+ slightly more functional)	All locations similar
KLRG1	Increased (progressive+)	Stable or –	Similar	Decreased	Opposite!: IR+ = more cytokines	No (iR+ slightly more functional)	All locations similar
BTLA	Decreased (progressive −)	Stable or +	Slightly increased	Slightly increased	Yes	No	All locations similar

*^a^For every + or − sign = a change of expression in 20% of cells can be seen*.

*Orange = positive or increase (dark = with at least one +); Blue = negative or decrease (dark = with at least one −)*.

*^b^Compared to blood*.

*^c^“Artifact” refers to the fact that the interpretation of functional differences in iR positive versus negative cells based on total cells is misleading, with an inherent differentiation bias. For example, in total cells, BTLA-cells are more functional because they are highly enriched for differentiated effector cells, and not because BTLA expression would be tightly associated with low functionality in those cells*.

*NA, not applicable*.

*Trend = non-significant tendency in this direction seen*.

## Discussion

Expression of iRs by CD8 T cells is generally considered a hallmark of “T cell exhaustion.” Particularly in the context of chronic antigen exposure, such as persistent viral infections and cancer, expression of iRs has been tightly linked with lower cytokine production.

The molecular mechanisms of T cell inhibition that are mediated by the various iRs, and how these integrate in the complex T cell signaling network, are only partially understood (1). Nevertheless, it is now recognized that T cell co-signaling is largely context dependent and relies on a diverse array of co-stimulatory and co-iRs that are spatiotemporally regulated and may have distinct or over-lapping functions in T cell priming, activation, differentiation, and memory responses (1). However, the relationships to these aspects (activation, differentiation, memory) remain as yet unknown or not determined for several iRs, particularly in human CD8 T cells (1).

In this study, we performed a broad analysis on associations between the expression of iRs and cytokine production in human CD8 T cells. We therefore assessed whether iRs are always markers of CD8 T cell dysfunction, by concisely analyzing whether CD8 T cells positive for a given iR were more or less functional (cytokine-productive) than their iR negative counterparts. There were sharp differences in the expression of iRs amongst the various differentiation subsets in human CD8 T cells (18). Cytokine production was also variable depending on the differentiation subset considered. Critically, we found that iR positive CD8 T cells are not necessarily less functional than their iR negative counterparts, but this may hold true provided that the differentiation stage is carefully taken into account. Subsets must be individually considered, or for the least naïve cells should be distinguished from non-naïve cells. Considering total cells, iR positive cells can appear more or less functional than their iR negative counterparts, but this is predominantly due to the inherent distinct expression of iRs and cytokines amongst the differentiation subsets.

Associations between iR expression and cytokine production were also investigated in the context of the TILN and non-metastatic LNs. Table 1 shows an overview on the different observations described for each iR. Prominently, differentiation and activation had the strongest impact on iR expression. Out of the eight iRs considered, five showed an increase and one a decrease with differentiation, and five showed an increase and two a decrease with activation (for full details refer to Table 1). In addition, both the CD8 T cells from metastatic and non-metastatic LN tissues showed differences in the expression of iRs and in cytokine production as compared to blood-derived CD8 T cells. Certain iRs (PD1, CTLA-4, TIM-3, LAG-3) can be particularly highly expressed in the tumor microenvironment (TILN), but there was high variability. These alterations in TILN are in agreement with previously described observations for TILN or tumor-infiltrating lymphocytes (TIL) material in melanoma patients (5, 19, 30), however, non-metastatic LN tissue had not been included as a control in these studies. Intriguingly, in previous work we had observed that CMV-specific CD8 T cells were less functional in metastatic as well as non-metastatic LN tissue, in addition to the observation that Melan-A-specific cells were impaired in melanoma lesions compared to blood, but can rapidly re-acquire function *ex vivo*. However, iR expression was not considered at that time [iRs and “T cell exhaustion” were not described in tumor immunology (17)]. Similarly, we more recently observed that EBV-specific CD8 T cells showed alterations in their iR profile in TILN compared to blood, similar to Melan-A specific CD8 T cells, pointing toward an anatomical influence on iR expression (5). Although we found comparable CD8 T cell dysfunction in TIL and TILN (17), it is possible that the non-lymphoid tumor microenvironment (i.e., TIL, enriched for tumor-specific CD8 T cells) presents more severe changes in iRs compared to TILN. Experimentally and clinically, it is much more difficult to obtain healthy tissue controls and TIL. Also, whether decreased cytokine production is due to the particular sample processing that is necessary with solid tissue material (from LNs and other tissues, but not with blood samples), particular subset distributions within LNs, or whether it reflects genuine decreased function in LNs, is difficult to assess. Nevertheless, our results comparing TILN versus normal (non-metastatic) LN suggest that altered iR and cytokine expression is not necessarily unique to the tumor microenvironment, but may be a general feature of lymphoid tissue compared to peripheral blood. High PD1 expression in CD8 T cells (and other iRs) may operate to limit self-tissue damage, similarly to the protection of vascular endothelium by the PD1:PDL1 axis as shown during viral infection (31).

Due to the low production of cytokines in TILN and normal LN, it was difficult to assess whether iR positive CD8 T cells are less functional than their iR negative counterparts. Nevertheless, in all tissues, a tendency toward less cytokines was found for non-naïve CD8 T cells that were PD1 positive or high, CTLA-4 positive, CD160 positive, while negative for 2B4 and KLRG1. For PD1, this is in agreement with previous findings using CD8 T cells from TIL material (19), as well as the functional deficiency seen particularly in T cells expressing high levels of PD1 (29).

Importantly, a direct link between positive iR expression and lower cytokine production was only found conclusively for CTLA4 and CD160, with only a tendency in this direction observed for PD1 (i.e., three of seven iRs considered, Table 1). Furthermore, there was substantial inter-donor variability (Figure S4 in Supplementary Material). Our data do not support that iRs can be considered general markers of decreased cytokine production, with this notion being the exception rather than the rule in our experiments. Instead, while anatomical location (LN versus blood) can influence the expression of iRs as well as cytokine production, our data clearly show that expression of iRs in human CD8 T cells is primarily dictated by T cell differentiation as well as activation.

Interestingly, several reports have already suggested that iR expression may not be directly or solely linked to impaired T cell function. Specifically, PD1 expression correlated with positive T cell function or activation markers such as 4-1BB in breast cancer and CD38 in HIV (14, 15, 32); the differentiation marker CD127 and not “exhaustion markers” correlated with positive prognosis in HCV infection (16); CD127 levels also correlated with PD1, 2B4, CD160, and KLRG1 expression in HCV infection (33); TIM3 expression correlated with effector memory phenotype in active tuberculosis (34), or questions were raised regarding PD1 as “exhaustion” marker in SIV infection depending on whether total or memory T cell were considered (35). While we find that BTLA exceptionally predominates in Naïve cells [Figure 1; (18, 23)], concordantly, BTLA expression in TIL used for adoptive therapy of melanoma patients is associated with better tumor regression (36). More recently, the “exhaustion marker” value of PD1 and BTLA has been alternatively reviewed in the context of both cancer and T cell differentiation (37). Also, the concern that PD1 is not necessarily a marker of T cell exhaustion was recently raised in the context of Acute Friend retrovirus infection, where CD8 T cells upregulate PD1 yet are highly cytotoxic and control virus (38). In the context of HIV infection, co-expression of PD1 and CD160 was also shown to discriminate between dysfunctional PD1hi CD8 T cells (PD1+ CD160+) versus CD8 T cells that up-regulated PD1 as a result of T cell activation (PD1+ CD160−) based on transcriptional profiling (39). Very recently, in the SIV infection model, the use of PD1 as a marker of exhaustion was questioned and found not be reliable when total T cells were considered (35). In fact, in the LCMV mouse model of “T cell exhaustion,” the evidence shows that iRs are strongly upregulated in the early phases of both acute and chronic LCMV infection in mice (12). Thereafter, only in the chronic setting are the iRs maintained or further increased, while CD8 T cells progress to resting memory in the acute setting. CD8 T cells derived from chronic or acute LCMV infections are fundamentally different in terms of their differentiation but also their activation status. Yet the activation, effector, and acute component of strong iR expression has been neglected in the consideration of the T cell “exhaustion” model (4, 40), and only recently and limitedly addressed (1, 8).

In addition, possible differences between the mouse and human systems must be carefully considered. In particular, KLRG1 does not correlate to PD1 expression in the exhaustion model of LCMV infection in mice (13). Interestingly, KLRG1 does exert inhibitory function in human CD8 T cells but not mouse cells (41, 42). The importance of KLRG1 in mouse “exhausted” CD8 T cells is unclear. It could be that KLRG1 expression does not correlate with CD8 T cell exhaustion or that KLRG1-expressing CD8 T cells were deleted during the chronic infection. We find that KLRG1 but also 2B4 [related to exhaustion in mice (13)] behaved very similarly in our experiments using human CD8 T cells, correlating strongly with more advanced T cell differentiation but poorly with lower cytokine production.

It is important to note that we used CD3- and CD28-specific antibodies for stimulation, coated on beads, at a 1:1 ratio with cells. Lower doses of stimulus (e.g., 0.2 beads per CD8 T cell) resulted in generally diminished cytokine production, which made it difficult to assess functionality in general. Nevertheless, comparing iR positive versus negative counterparts, the trends were very similar at lower and higher bead-to-cell ratio (data not shown). Notably, we did not use high doses of beads nor other overwhelming activatory treatments such as PMA and ionomycin. This polyclonal setup allowed, on the one hand, to optimally activate all subsets and all cells independently of TCR specificity and functional avidity. On the other hand, and critically, this broad T cell stimulus allowed us to concisely assess and compare the functional potential of iR positive versus iR negative CD8 T cells. This setup, however, does not address the function nor immediate influence of iRs *per se on* T cell function, differentiation, or activation. Moreover, the notion that differentiation and activation primarily drive iR expression is well compatible with the concept that iR–iR Ligand interactions can negatively interfere with CD8 T cell function. Our experiments did not address and our results do not exclude that iRs, triggered by their ligands, inhibit CD8 T cells. There is no doubt that iR positive cells can be inhibited by stimulator or target cells expressing their ligands, when interacting antigen-specifically in the context of a physiological immune synapse (1, 43–45).

In chronic infection and cancer, iRs contribute to T cell inhibition and the stumbling blocks faced by T cell-based immunotherapies (44). Preclinical and clinical studies have demonstrated the usefulness of treatments with antibodies blocking iRs (46). For the further development of such therapies, it is therefore important to monitor iR expression and function of CD8 T cells, together with the differentiation and activation status of the cells. We find that iR positive CD8 T cells are not necessarily dysfunctional, but can be more or less differentiated. Moreover, we showed a dramatic up-regulation of certain iRs during T cell stimulation, following the peak of cytokine production, and in tight positive correlation with several activation markers. This emphasizes the notion that expression of multiple iRs can be due to recent or ongoing CD8 T cell activation, and that expression of iRs may in fact mark the cells that responded best to a given stimulus.

Interestingly, positive PDL1 expression in tumors is a good prognostic indicator in some cancers, such as melanoma (47), reflecting ongoing CTL responses (48) and better chances of successful anti-PD1 therapy (49). In turn, PD1 is increased in Melan-A-reactive CD8 T cells with progression of melanoma, although the prognostic value of PD1 on CD8 T cells is less clear, with no association to overall survival in melanoma or a positive prognostic value in other types of cancers such as HPV-induced head and neck cancer (50, 51). Using the prototypic LCMV mouse model of T cell exhaustion, we recently showed that CD8 T cells from chronic infection retain the “exhaustion” phenotype upon transfer to naïve mice yet are capable of re-expansion and protection under re-challenge with acute LCMV infection (25). Within this latter study, we already reported that PD1 positive CD8 T cells in PBMC from healthy donors or melanoma patients are not necessarily functionally impaired. In this study, we broaden the observations to several iRs, in healthy donors and patients, studying the link between iR expression and cytokine production, and critically, considering activation, differentiation as well as anatomical location. Altogether, these results and the aforementioned literature points toward a context-dependent expression of iRs and that many “exhausted” or iR positive CD8 T cells retain functional capacity, in support of the immunotherapeutic potential of blocking iRs. In controlled experimental systems where the iR ligands are present (29, 52, 53), complement studies will analyze the functional consequence of blocking one or several iR–iR ligand interactions.

Our present observations using human CD8 T cells highlight that iRs are often misinterpreted as only “exhaustion markers,” in a “guilty by association” reflex. While there is evidence for a clear correlation between increased iRs and lower cytokine potential in CD8 T cells under chronic antigen exposure (viral infection and cancer), our data show that the direct link between iR expression and lower cytokines is weak. Rather, activation and differentiation are strong primary drivers of iR expression, and both factors should be carefully taken into account within the study and clinical monitoring of normal and pathological CD8 T cell functions.

## Author Contributions

Conceived and designed the experiments: Amandine Legat, Daniel E. Speiser, Hanspeter Pircher, Dietmar Zehn, Silvia A. Fuertes Marraco. Performed the experiments: Amandine Legat, Silvia A. Fuertes Marraco. Analyzed the data: Amandine Legat, Silvia A. Fuertes Marraco. Wrote the paper: Amandine Legat, Daniel E. Speiser, Hanspeter Pircher, Silvia A. Fuertes Marraco.

## Conflict of Interest Statement

The authors declare that the research was conducted in the absence of any commercial or financial relationships that could be construed as a potential conflict of interest.

## Supplementary Material

The Supplementary Material for this article can be found online at http://www.frontiersin.org/journal/10.3389/fimmu.2013.00455/abstract

Figure S1 **Gating strategy for the analysis of human CD8 T cells**. **(A)**. Gating of total live CD8 T cells, based on size, doublet exclusion, and finally selection of CD8+, Vivid (dead) negative and CD16 (NK marker) negative. **(B)**. Gating of the various differentiation subsets based on CD45RA and CCR7, as indicated.

Figure S2 **Non-naïve CD8 T cells show poly functionality, in contrast to the absence of cytokines and CD107a in Naïve cells**. **(A)**. Representative plots showing CCR7 versus CD45RA in total CD8 T cells as well as IFNg and TNFa within the various subsets during the 24 h kinetics of stimulation with aCD3 and aCD28 beads. **(B)**. Quantification (% positive) of the production of IFNg, TNFa, IL-2, and Granzyme B as well as CD107a translocation in the various CD8 T cell subsets from healthy donors stimulated for 6 h with aCD3 and aCD28 beads (*N* = 5). These data correspond to the samples and complement the data shown in Figure 2.

Figure S3 **Additional data on the dynamics of iR expression and correlation to cytokine production depending on differentiation (in complement to Figure 2B)**. The analysis described in Figure 2B is additionally detailed for the various differentiation subsets (*N* = 5).

Figure S4 **Additional data on the cytokine production in iR positive versus iR negative counterparts, depending on CD8 T cell differentiation (in complement to Figure 4)**. The data described in Figure 4 is analyzed for total cytokine-producing cells (a single parameter based on the % of IFNg and/or TNFa positive cells, *N* = 15). Pairing lines indicate iR positive and negative counterparts within the same sample and gate. *p*-Values are based on repeated measures ANOVA with Bonferroni correction.

Figure S5 **Additional data on the subset distribution as well as correlation of various iRs and cytokines to the proportion of CM in the various sample groups (in complement to Figure 6)**. The experiment and samples described in Figure 6 were analyzed for subset distribution, shown in **(A)**. Further analyses addressed the relationship between the proportion of CM cells in the various sample groups and the percentage of iR positive **(B)** or cytokine positive cells **(C)**. *p*-Values in brackets indicate Pearson correlations, performed either on all samples or within individual groups of samples (indicated in each graph’s legend). NA, not applicable, because samples fall below the detection limit of 1%.

Figure S6 **High expression of PD1 cells can be found in metastatic (TILN) and non-metastatic LN, in contrast to blood (in complement to Figure 6)**. The experiment from Figure 6 was analyzed in particular detail for PD1 expression in Non-Naïve cells. **(A)**. PD1 versus FS-area plot of Non-naive CD8 T cells are shown to render the PD1hi population more visible as compared to the off-set overlays shown in Figure 6A. One representative example is shown for each, i.e., PBMC of healthy donor, PBMC of melanoma patient and non-metastatic LN, and two distinct examples are shown for TILN. **(B)**. Quantification showing the percentage positive cells for PD1 or PD1 high, in the form of dotted plots to see the samples below 1% that are excluded for the analysis in C. *p*-Values were based on multiple comparisons using one-way ANOVA with Bonferroni correction. **(C)**. Analysis of the expression of TNFa and IFNg in PD1 high, intermediate or negative cells, within non-naïve cells, amongst the indicated LN groups. Pie arcs and slices represent the percentage positive for IFNg and/or TNFa as indicated in the legend.
