# Supplementary figures and images for: Inhibitory Receptor Expression Depends More Dominantly on Differentiation and Activation than “Exhaustion” of Human CD8 T Cells
Source: Front Immunol. 2013 Dec 19;4:455. doi: 10.3389/fimmu.2013.00455 (PMC3867683; doi:10.3389/fimmu.2013.00455)

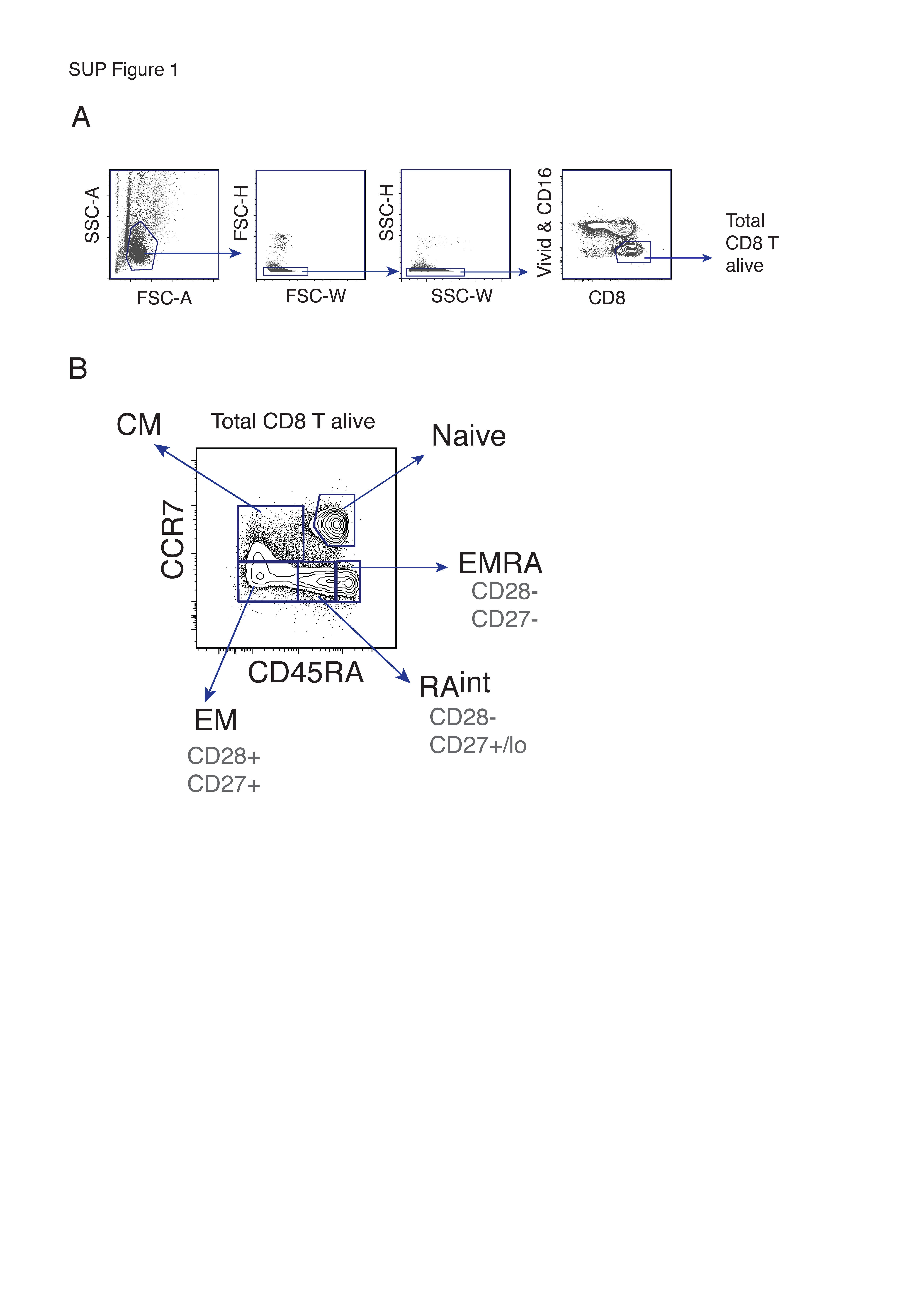

Supplement: Figure S1 — Gating strategy for the analysis of human CD8 T cells. (A). Gating of total live CD8 T cells, based on size, doublet exclusion, and finally selection of CD8+, Vivid (dead) negative and CD16 (NK marker) negative. (B). Gating of the various differentiation subsets based on CD45RA and CCR7, as indicated. [file 66759_Speiser_DataSheet1.ZIP › 66759__Data_Sheet_1 2/66759_Speiser_Figure_S1.TIFF]

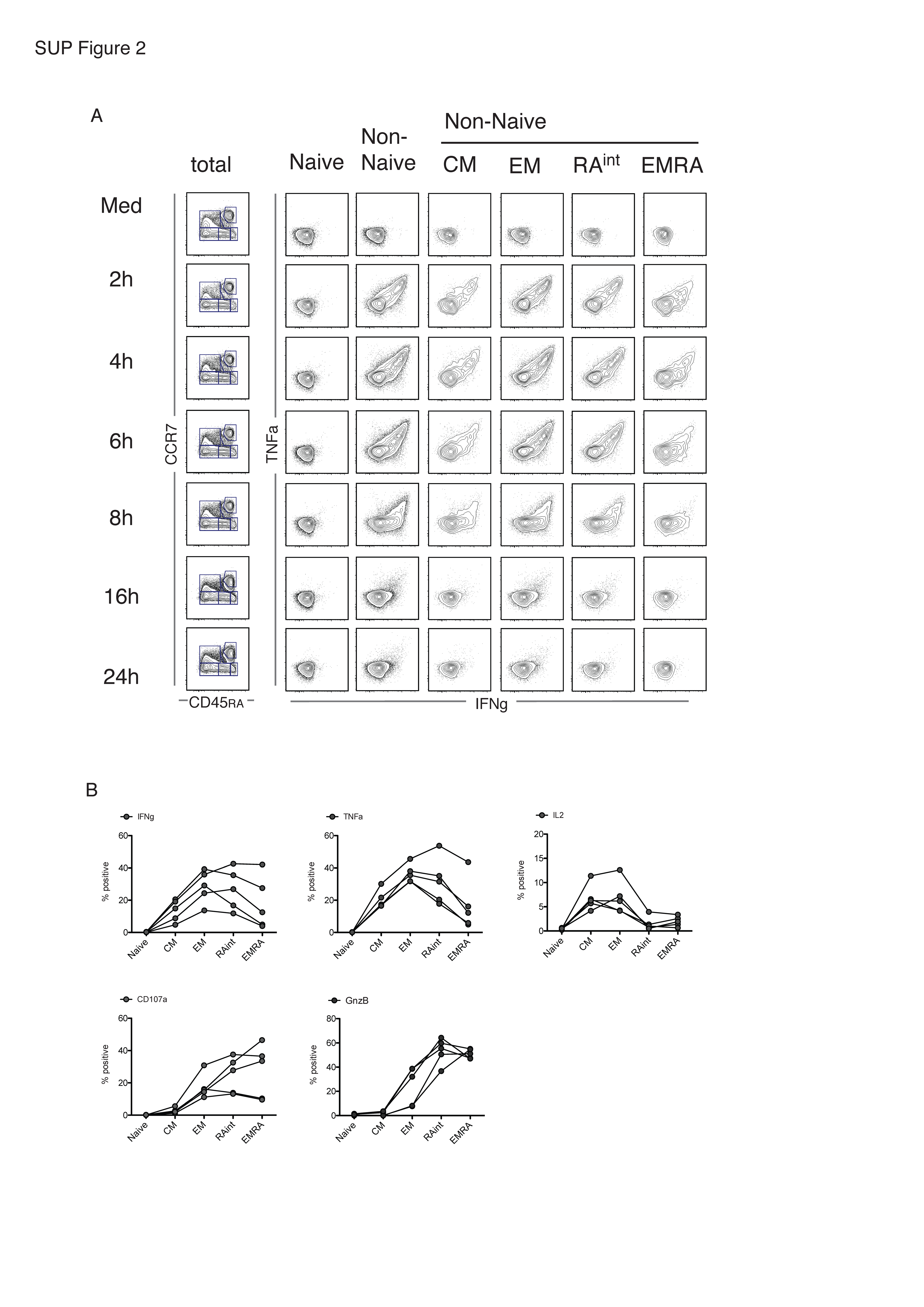

Supplement: Figure S1 — Gating strategy for the analysis of human CD8 T cells. (A). Gating of total live CD8 T cells, based on size, doublet exclusion, and finally selection of CD8+, Vivid (dead) negative and CD16 (NK marker) negative. (B). Gating of the various differentiation subsets based on CD45RA and CCR7, as indicated. [file 66759_Speiser_DataSheet1.ZIP › 66759__Data_Sheet_1 2/66759_Speiser_Figure_S2.TIFF]

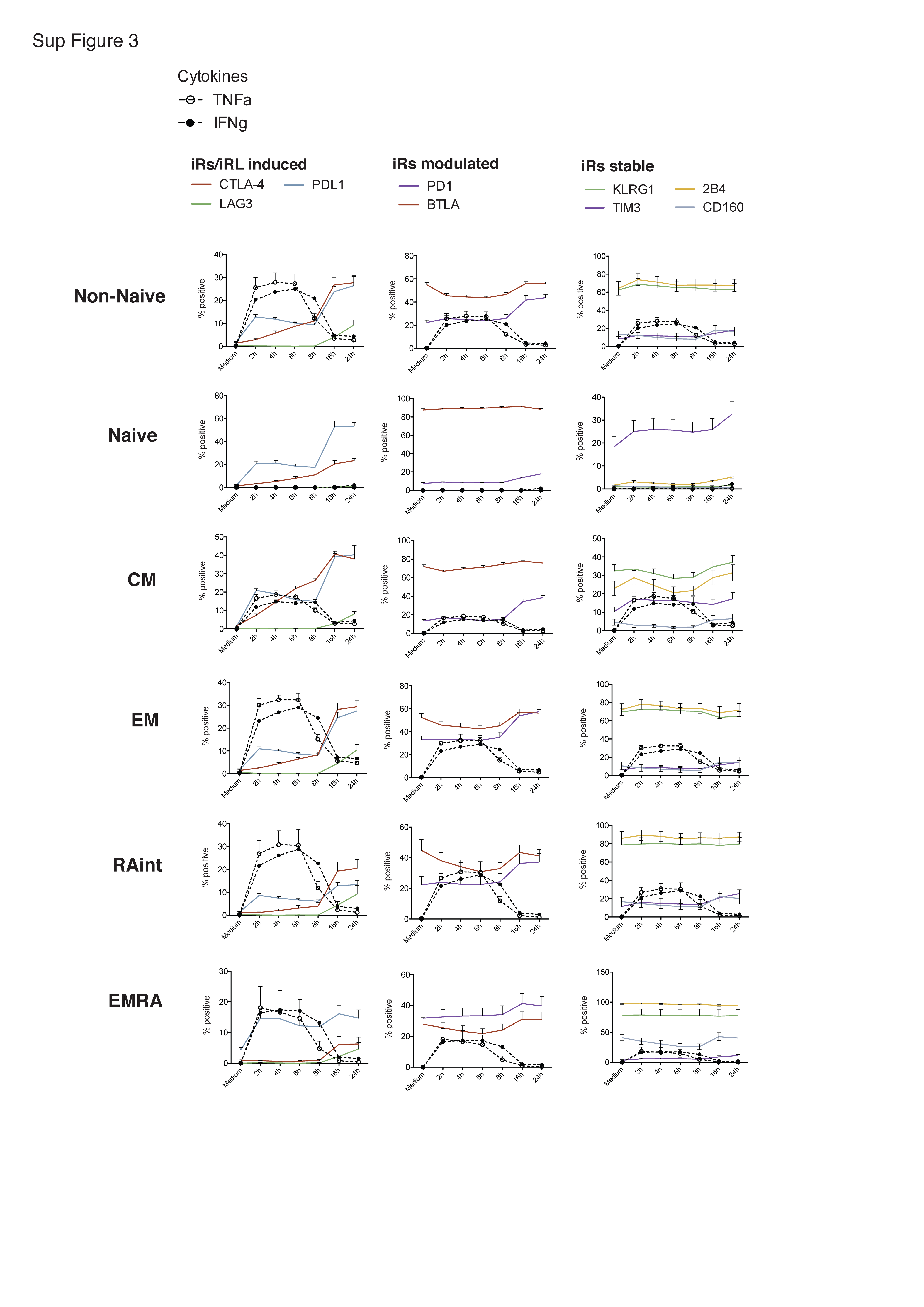

Supplement: Figure S1 — Gating strategy for the analysis of human CD8 T cells. (A). Gating of total live CD8 T cells, based on size, doublet exclusion, and finally selection of CD8+, Vivid (dead) negative and CD16 (NK marker) negative. (B). Gating of the various differentiation subsets based on CD45RA and CCR7, as indicated. [file 66759_Speiser_DataSheet1.ZIP › 66759__Data_Sheet_1 2/66759_Speiser_Figure_S3.TIFF]

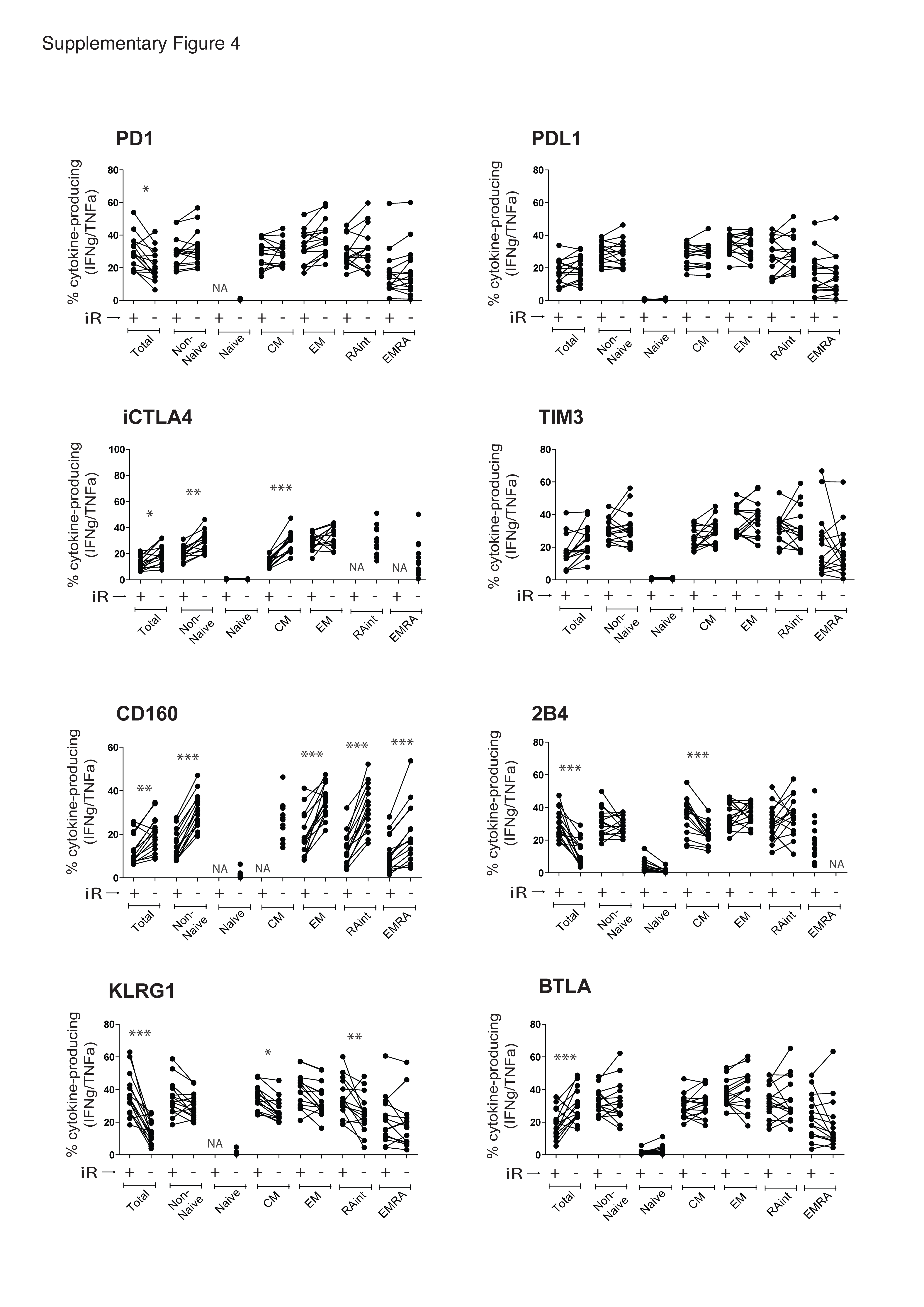

Supplement: Figure S1 — Gating strategy for the analysis of human CD8 T cells. (A). Gating of total live CD8 T cells, based on size, doublet exclusion, and finally selection of CD8+, Vivid (dead) negative and CD16 (NK marker) negative. (B). Gating of the various differentiation subsets based on CD45RA and CCR7, as indicated. [file 66759_Speiser_DataSheet1.ZIP › 66759__Data_Sheet_1 2/66759_Speiser_Figure_S4.TIFF]

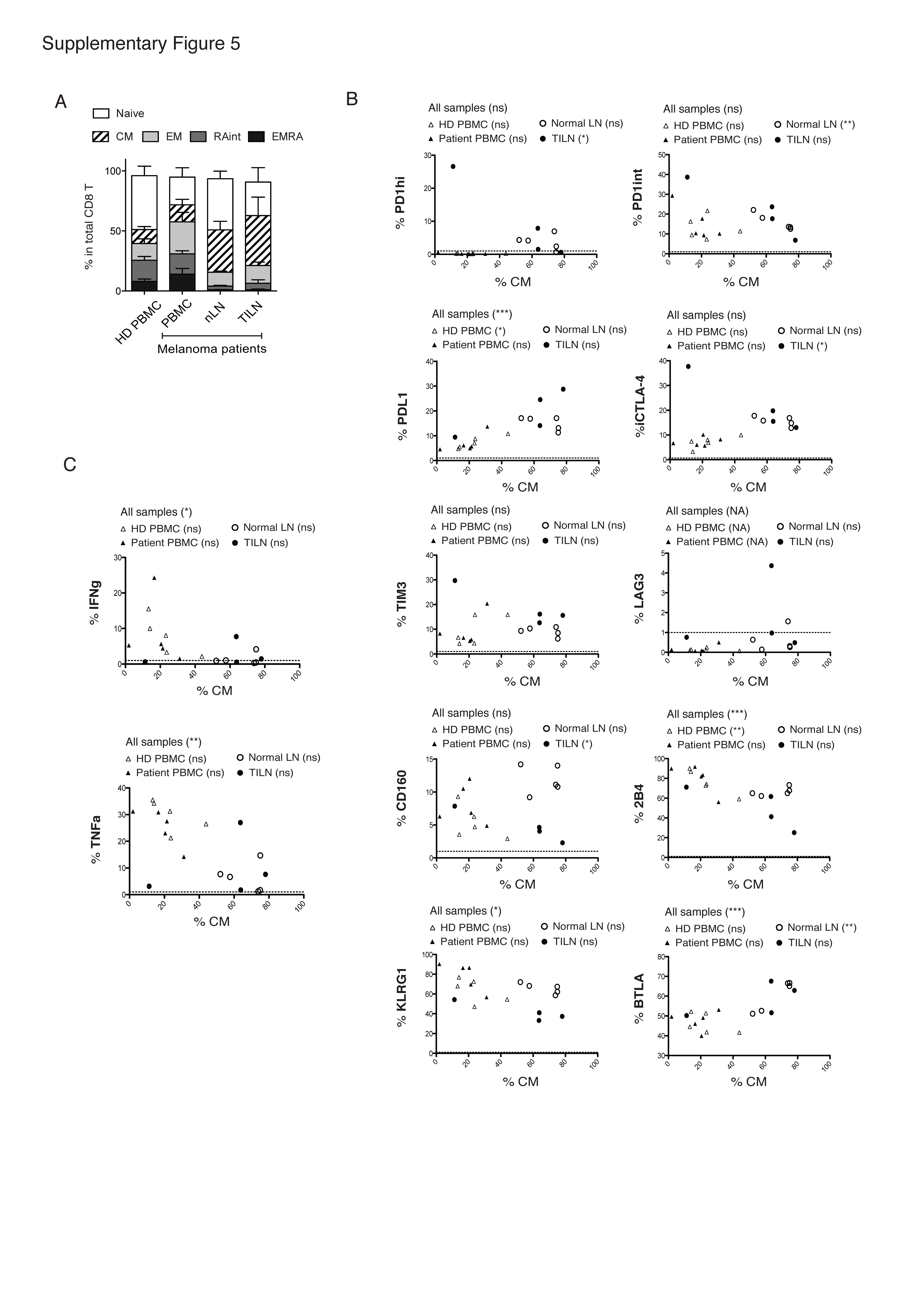

Supplement: Figure S1 — Gating strategy for the analysis of human CD8 T cells. (A). Gating of total live CD8 T cells, based on size, doublet exclusion, and finally selection of CD8+, Vivid (dead) negative and CD16 (NK marker) negative. (B). Gating of the various differentiation subsets based on CD45RA and CCR7, as indicated. [file 66759_Speiser_DataSheet1.ZIP › 66759__Data_Sheet_1 2/66759_Speiser_Figure_S5.TIFF]

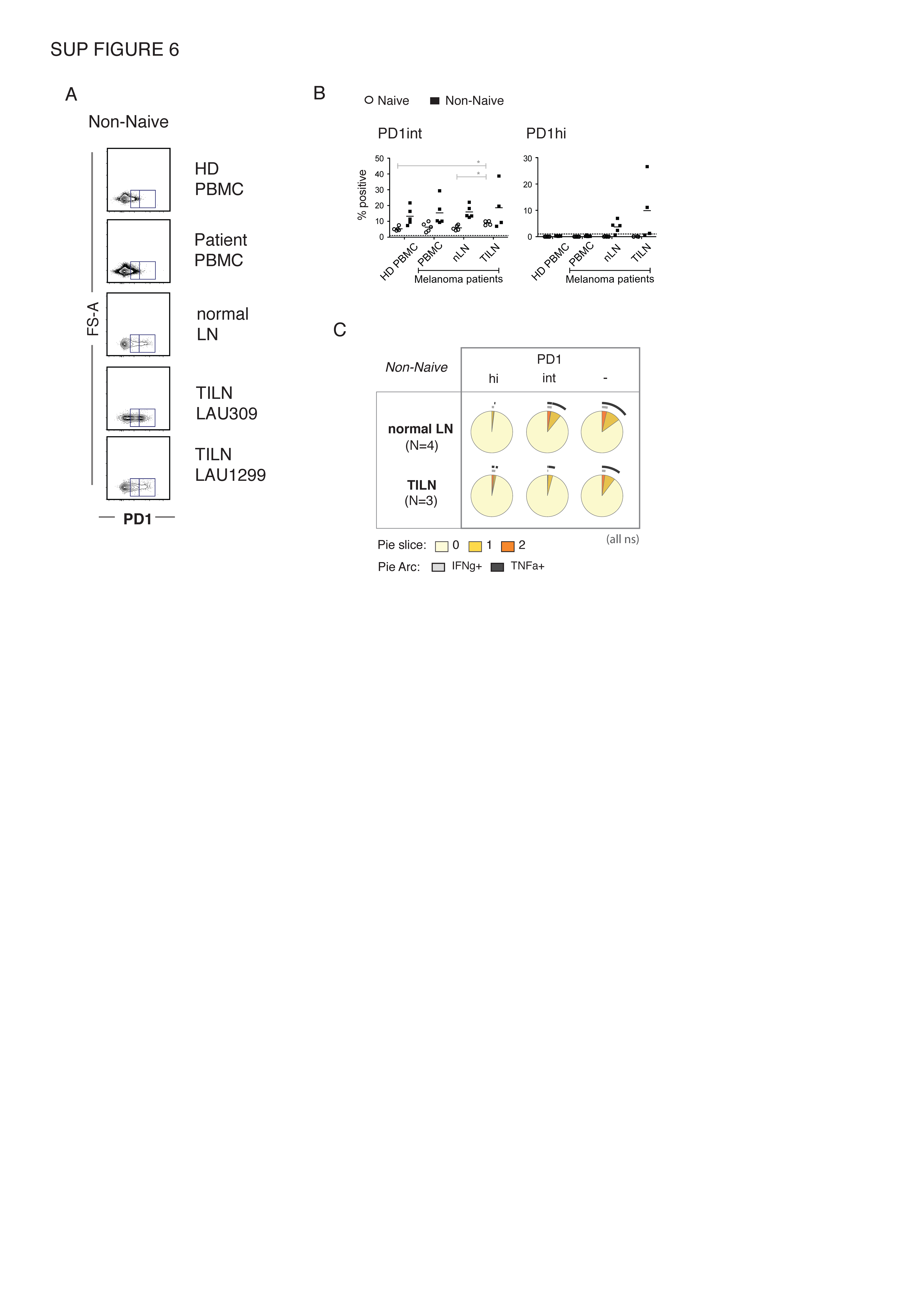

Supplement: Figure S1 — Gating strategy for the analysis of human CD8 T cells. (A). Gating of total live CD8 T cells, based on size, doublet exclusion, and finally selection of CD8+, Vivid (dead) negative and CD16 (NK marker) negative. (B). Gating of the various differentiation subsets based on CD45RA and CCR7, as indicated. [file 66759_Speiser_DataSheet1.ZIP › 66759__Data_Sheet_1 2/66759_Speiser_Figure_S6.TIFF]
